# L-type amino acid transporter (LAT) 1 expression in ^18^F-FET-negative gliomas

**DOI:** 10.1186/s13550-021-00865-9

**Published:** 2021-12-14

**Authors:** Franziska J. Vettermann, Caroline Diekmann, Lorraine Weidner, Marcus Unterrainer, Bogdana Suchorska, Viktoria Ruf, Mario Dorostkar, Vera Wenter, Jochen Herms, Jörg-Christian Tonn, Peter Bartenstein, Markus J. Riemenschneider, Nathalie L. Albert

**Affiliations:** 1 Department of Nuclear Medicine, University Hospital of Munich, LMU Munich, Munich, Germany; 2 Department of Neuropathology, Regensburg University Hospital, Regensburg, Germany; 3 Department of Radiology, University Hospital of Munich, LMU Munich, Munich, Germany; 4 Department of Neurosurgery, University Hospital of Munich, LMU Munich, Munich, Germany; 5 Present Address: Department of Neurosurgery, Sana Hospital, Duisburg, Germany; 6 Center for Neuropathology, University Hospital of Munich, LMU Munich, Munich, Germany; 7 German Cancer Consortium (DKTK), Partner Site Munich, German Cancer Research Center (DKFZ), Heidelberg, Germany

**Keywords:** LAT1, FET PET, Glioma, Molecular imaging

## Abstract

**Background:**

O-(2-[^18^F]-fluoroethyl)-L-tyrosine (^18^F-FET) is a highly sensitive PET tracer for glioma imaging, and its uptake is suggested to be driven by an overexpression of the L-type amino-acid transporter 1 (LAT1). However, 30% of low- and 5% of high-grade gliomas do not present enhanced ^18^F-FET uptake at primary diagnosis (“^18^F-FET-negative gliomas”) and the pathophysiologic basis for this phenomenon remains unclear. The aim of this study was to determine the expression of LAT1 in a homogeneous group of newly diagnosed ^18^F-FET-negative gliomas and to compare them to a matched group of ^18^F-FET-positive gliomas. Forty newly diagnosed *IDH*-mutant astrocytomas without 1p/19q codeletion were evaluated (n = 20 ^18^F-FET-negative (tumour-to-background ratio (TBR) < 1.6), n = 20 ^18^F-FET-positive gliomas (TBR > 1.6)). LAT1 immunohistochemistry (IHC) was performed using SLC7A5/LAT1 antibody. The percentage of LAT1-positive tumour cells (%) and the staining intensity (range 0–2) were multiplied to an overall score (H-score; range 0–200) and correlated to PET findings as well as progression-free survival (PFS).

**Results:**

IHC staining of LAT1 expression was positive in both, ^18^F-FET-positive as well as ^18^F-FET-negative gliomas. No differences were found between the ^18^F-FET-negative and ^18^F-FET-positive group with regard to percentage of LAT1-positive tumour cells, staining intensity or H-score. Interestingly, the LAT1 expression showed a significant negative correlation with the PFS (*p* = 0.031), whereas no significant correlation was found for TBR_max_, neither in the overall group nor in the ^18^F-FET-positive group only (*p* = 0.651 and *p* = 0.140).

**Conclusion:**

Although LAT1 is reported to mediate the uptake of ^18^F-FET into tumour cells, the levels of LAT1 expression do not correlate with the levels of ^18^F-FET uptake in *IDH*-mutant astrocytomas. In particular, the lack of tracer uptake in ^18^F-FET-negative gliomas cannot be explained by a reduced LAT1 expression. A higher LAT1 expression in *IDH*-mutant astrocytomas seems to be associated with a short PFS. Further studies regarding mechanisms influencing the uptake of ^18^F-FET are necessary.

**Supplementary Information:**

The online version contains supplementary material available at 10.1186/s13550-021-00865-9.

## Background

Positron emission tomography (PET) with radiolabelled amino acids, such as ^18^F-fluoro-ethyl-tyrosine (^18^F-FET), is increasingly implemented in the clinical routine of glioma diagnosis and used for non-invasive tumour grading, biopsy guidance and for surgery or radiotherapy planning [1]. Several studies suggest that the uptake of ^18^F-FET is primarily promoted by the L-amino acid transporter 1 (LAT1), coding by solute carrier family 7 member 5 (SLC7A5), though this has not yet been conclusively proven [2–7]. So far it is hypothesized that on a cellular basis, the radiolabelled amino acid analogue ^18^F-FET is predominantly taken up via upregulated LAT1 into the tumour cell. Its accumulation is mediated by the asymmetric recognition of the amino acid derivative at the two sides of the cell membrane, inducing a trapping mechanism without incorporation into proteins or metabolization [7]. The main function of the LAT1 protein is to help specific amino acids pass through the cell membrane to provide nutrients to cells and participate in metabolic pathways. LAT1 requires another cell surface glycoprotein, the 4F2 heavy chain (4F2hc), for its functional expression, and recent studies describe the importance for transport activity [8]. Together both proteins form a heterodimeric transport complex [9]. It has previously been described that the light chain LAT1 and 4F2hc expression is positively correlated and it is assumed that the light chain LAT1 expression level corresponds to that of 4F2hc [10].

Besides being an ubiquitous Na+- and H+-independent antiporter, involved in cellular uptake of essential amino acids, the L-type amino acid transporter 1 (LAT1) is over-expressed in many human cancer cells that are characterized by an increased demand of essential amino acids, including gliomas [11–15]. The upregulated expression of LAT1 may benefit by providing tumour cells with essential amino acids for high levels of protein synthesis associated with cell activation to support rapid growth or excessive proliferation. LAT1 has been described as a prognostic marker for malignant progression and proliferation of high-grade gliomas and correlates closely with the glioma angiogenesis [10, 16].

However, approximately 30% of low-grade glioma and 5% of high-grade glioma do not present an enhanced ^18^F-FET uptake at primary diagnosis. During follow-up and progression, around one-half of these ^18^F-FET-negative gliomas (tumour-to-background ratio, TBR < 1.6) develop an increased ^18^F-FET uptake (TBR > 1.6) [17]*.*

While the pathophysiologic mechanisms leading to ^18^F-FET uptake and the different dynamic uptake characteristics are not yet fully clarified, it particularly remains uncertain which cellular mechanisms lead to the phenomenon of missing intracellular ^18^F-FET uptake in ^18^F-FET-negative gliomas. Recent studies have described a third subset of gliomas with an ^18^F-FET uptake even below the physiological brain tissue uptake (so-called photopenic gliomas), which are associated with a worse prognosis [17–19].

In need for a better understanding of the missing ^18^F-FET uptake in ^18^F-FET-negative gliomas, we evaluated the LAT1 expression levels in a homogeneous group of newly diagnosed *IDH*-mutant astrocytomas (without 1p/19q codeletion) and compared them with a neuropathologically and sex-matched ^18^F-FET-positive glioma group and correlated *in vivo* and *in vitro* parameters with clinical survival data.

## Methods

### Patients

Forty newly diagnosed, histologically verified *IDH*-mutant gliomas WHO grade II and III without 1p/19q codeletion (according to the 2016 WHO classification) with preoperative ^18^F-FET PET were included in this retrospective study. Forty-minutes dynamic ^18^F-FET PET scans were acquired with an ECAT EXACT HR + scanner (Siemens Healthcare) according to standard protocols after a slow intravenous bolus injection of approximately 180 MBq of ^18^F-FET.

All patients were therapy-naive before surgical resection or biopsy. All specimens were obtained at initial surgery. Patients included 17 females and 23 males with a median age of 38.2 years (range 25.9–69.3 years). Thirty-one patients were diagnosed with a WHO grade II and 9 cases with a WHO grade III glioma. All patients had given written consent prior to the ^18^F-FET PET scan as part of the clinical routine. The present analysis was approved by the local ethics committee (606-16).

### PET acquisition and mode of evaluation

Dynamic ^18^F-FET PET scans (40 min; 16 frames) were acquired with an ECAT Exact HR + scanner (Siemens) according to standard protocols and evaluated on a Hermes workstation (Hermes Medical Solutions) as described previously [20]. For the assessment of the maximal tumour-to-background ratio (TBR_max_) and the mean tumour-to-background ratio (TBR_mean_), the maximal and mean standardized uptake value (SUV_max,_ SUV_mean_) of the tumour was corrected for the mean background activity in the healthy contralateral hemisphere.

Furthermore, the biological tumour volume (BTV) was estimated by semiautomatic calculation of a volume of interest using a threshold of TBR ≥ 1.6, which has been proposed as the optimal threshold between tumour and surrounding healthy tissue [21]. Accordingly, the mean tumour-to-background ratio (TBR_mean_) was evaluated as the mean standardized uptake (SUV_mean_) within the BTV divided by the mean background activity in the healthy contralateral hemisphere.

Dynamic PET recordings were evaluated according to our standardized clinical procedure as described previously [22]. Within the 40-min dynamic scan, time-to-peak (TTP) was assessed in each slice within the tumour and consequently the shortest TTP in at least two consecutive slices was defined as minimal TTP (TTP_min_) [23]. Regarding the exclusion of noise artefacts in the beginning of the PET acquisition due to low counting rates, only frames 11–16 (3–40-min p.i.) were analysed in the dynamic evaluation. According to the length of our frames, TTP_min_ is appointed for 4, 7.5, 12.5, 17.5, 25 and 35 min in frames 11–16, respectively.

SUV_mean_, TBR_mean_, BTV and TTP_min_ could only be assessed in the ^18^F-FET-positive group since the semiautomatic derived output is dependent on the threshold (TBR ≥ 1.6) which is not reached in ^18^F-FET-negative gliomas (TBR < 1.6).

Tumours were classified as ^18^F-FET–positive, if an increased ^18^F-FET uptake above cerebral background activity was observed (TBR > 1.6); consequently, they were rated as ^18^F-FET-negative if tumours were not delineated from cerebral background activity in the PET scans (TBR < 1.6). For a further subgroup analysis ^18^F-FET-photopenic lesions with ^18^F-FET uptake below background activity (TBR < 1.0) were defined.

### Neuropathological assessment

Histological assessment was carried out on routine histological sections after formalin fixation and paraffin embedding of the biopsy specimens. Tumour type and malignancy grade of each tumour were determined according to the 2016 World Health Organization classification of tumours of the nervous system using conventional staining (haematoxylin and eosin, reticulin stain), immunohistochemistry and molecular markers [24].

### Immunohistochemistry for LAT1

Immunohistochemical staining was performed according to standard protocols [25, 26]. Briefly, 3-μm-thick paraffin sections were deparaffinized and rehydrated. For LAT1 immunostaining, antigen retrieval was performed in 10 mM citrate buffer (pH 6.0) in a microwave oven (900 W) for 30 min (3 × 10 min). After the sections were rinsed with the washing buffer (PBS, pH 7.4 + 0.05% Tween 20), the endogenous peroxidases were blocked for 10 min with the endogenous enzyme block from the EnVision™ + Dual Link System-HRP Kit (Dako by Agilent Technologies, Santa Clara, CA, USA). The kit was utilized also for the following steps according to the manufactures protocol. Incubation with the primary antibody anti-LAT1 (NBP2-33662) was done at a 1:100 dilution for 1 h at room temperature. After rinsing in washing buffer, labelled Polymer-HRP complex was added, and the sections were incubated for 30 min at room temperature. After another washing step, the peroxidase reaction was performed using 3,3'-diaminobenzidine tetrahydrochloride (DAB) in substrate buffer for 4 min at room temperature. The sections were then counterstained with haematoxylin, dehydrated and coverslipped.

### Scoring of immunohistochemical LAT1 staining

The immunohistochemical LAT1 staining was scored by a blinded, experienced neuropathologist (M.R.) to limit unintentional or subjective observation biases. The scorings were performed twice, each in one session. In case of stereotactic biopsies, scoring represents the whole tissue sections (stereotactic biopsy ~ 1 mm), and in cases of resections an average score from five random × 10 objective microscopic fields was used.

The H-score assigns an ordinal score to the immunostaining intensity and multiplies this by an estimate of the percentage of immunostained tissue for each intensity grade to assess the extent of immunoreactivity. The score is obtained by the formula: 2 × the percentage of strong staining + 1 × the percentage of moderate staining + 0 × the percentage of weak or absent staining [27]. The H-score was slightly adjusted in this study due to only three discriminable staining intensities in the LAT1 staining, resulting in scores ranging from 0 to 200.

The H-score was assessed for the overall tissue (LAT1 overall H-score), for tumour cells only (LAT1 H-score tumour cells) and for the staining of vessels only (LAT1 H-score vessels). Visual allocation of the LAT1 staining to either tumour cells or vessels was performed by an experienced neuropathologist (M.R.). In 10 out of 40 samples, no vessels were present.

### Evaluation of outcome

Progression-free survival (PFS) was defined as the time in months between initial diagnosis and first tumour progression according to the RANO criteria [28, 29]. The overall survival was not evaluable due to a high number of censored patients (low number of events and patients lost-to-follow up).

### Statistical analysis

Statistical analysis was performed with SPSS Statistics (version 25; IBM). Data were tested for normality of distribution using the Shapiro–Wilk test. Pearson product moment correlation was used to identify significant correlations between ^18^F-FET PET uptake and H-score of LAT1 expression. Cox regression analyses were performed to correlate PFS with LAT1 expression and ^18^F-FET uptake intensity. Patients were sorted into two groups by the respective median split of the above-described LAT1 overall H-score and TBR_max_ in order to perform Kaplan–Meier analyses with log-rank test. Statistical significance was defined for 2-tailed *p *values below 0.05.

## Results

### Patient’s characteristics

Twenty out of forty were classified as ^18^F-FET-negative (9 females, 11 males; median age 37.8 years, range 26.3–47.6 years; 5/20 ^18^F-FET-photopenic) and the other 20 patients as ^18^F-FET-positive (8 females, 12 males; median age 39.9 years, range 25.9–69.3 years). Further details are given in Table 1 and Additional file 1: Table S1.

**Table 1:** Demographics at group level

			^18^F-FET positive	^18^F-FET negative
Number of subjects			20	20
Age (y, median, range)			39.9 (25.9–69.3)	37.8 (26.3–47.6)
Sex (♀ /♂)			8♀/12♂	9♀/11♂
WHO grade	II		11	20
	III		9	0
Mode of surgery	Biopsy		14	19
	Resection		6	1
SUV_BG_ (median, range)			1.04 (0.67–1.93)	1.01 (0.35–1.20)
SUV_max_ (median, range)			2.91 (1.32–5.75)	1.16 (0.40–1.81)
TBR_max_ (median, range)			2.29 (1.61–5.17)	1.24 (0.84–1.58)
SUV_mean_ (median, range)			1.94 (1.19–3.09)	
TBR_mean_ (median, range)			1.93 (1.68–2.38)	
BTV (median, range)			19.52 (1.38–78.46)	
% LAT1-positive tumour cells by staining intensity (median, range)		0	70 (0–98)	80 (0–98)
		1	20 (0–95)	10 (0–95)
		2	5 (0–80)	5 (0–50)
LAT1 overall H-score (median, range)			22.5 (2–180)	25.5 (5–118)
LAT1 H-score tumour cells (median, range)			11.0 (0–180)	10.5 (0–84)
LAT1 H-score vessels (median, range)			6.5 (0–42)	4.5 (0–22)

SUV_max_ = mean background uptake; SUV_max_ = maximal standardized uptake value; TBR_max_ = maximal tumour-to-background ratio; SUV_mean_ = mean standardized uptake value (in ^18^F-FET-positive lesions only); TBR_mean_ = mean tumour-to-background ratio (in ^18^F-FET-positive lesions only); BTV = biological tumour volume (in ^18^F-FET-positive lesions only); %LAT1-positive tumour cells by staining intensity show the percentage of cells with no (0), low (1), or high (2) staining intensity within tissue samples in the two groups of ^18^F-FET-positive and ^18^F-FET-negative gliomas

With regard to the MRI, mostly minimal contrast enhancement was present in 11 gliomas and out of the nine WHO grade III gliomas only five showed contrast enhancement. All contrast-enhanced gliomas were ^18^F-FET-positive, but no correlation of TBR_max_ and the LAT1 expression was found.

### Static ^18^F-FET PET parameters

Static ^18^F-FET PET parameters were evaluated for ^18^F-FET-negative and ^18^F-FET-positive gliomas separately. The median TBR_max_ in the ^18^F-FET-negative group amounts to 1.24 (range 0.84–1.58), while the ^18^F-FET-positive group had a significantly higher median TBR_max_ of 2.29 (range 1.61–5.17; p < 0.001). ^18^F-FET-positive gliomas exhibited a TBR_mean_ of 1.93 (range 1.68–2.38) (Table 1).

### Dynamic ^18^F-FET PET parameters

^18^F-FET uptake dynamics were evaluated in ^18^F-FET-positive cases only (n = 20). In 2 patients dynamic data were not available. Nine gliomas showed an increasing time activity curve and nine gliomas a decreasing time activity curve. The median TTP_min_ in the ^18^F-FET-positive group was 30.0 min (range 12.5–35.0 min).

### Analyses of LAT1 expression

**Fig. 1:**
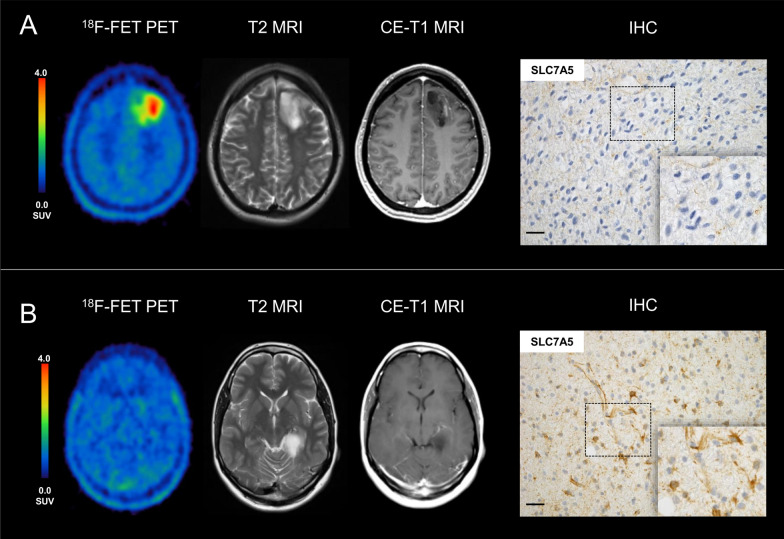
Patient example with a **A** ^18^F-FET-positive, WHO grade II glioma with a high ^18^F-FET-uptake (TBR_max_ 4.36) and small nodular contrast enhancement on MRI but with very low LAT1 tumour cell expression (H-score 9.0) and slightly positive vessels in the SLC7A5 immunohistochemical (IHC) staining in contrast to a **B** ^18^F-FET-negative, WHO grade II glioma (TBR_max_ 1.27) with T2 alterations and missing contrast enhancement on MRI but a very high LAT1 expression (H-score 102.0) and intensive vessel staining

IHC staining of LAT1 was positive in all specimens studied, both in ^18^F-FET-positive and ^18^F-FET-negative gliomas likewise. The small subset of ^18^F-FET-photopenic lesions (n = 5), lesions with a lower ^18^F-FET uptake compared to the background, was also visually positive for LAT1 staining. All tumour probes examined showed specific LAT1 expression regardless of their ^18^F-FET uptake level (Fig. 1). Overall, there was no significant difference in the amount of positive tumour cells and in the staining intensity (as semiquantitatively assessed by the H-Score) between ^18^F-FET-negative and ^18^F-FET-positive gliomas (minimal *p *value = 0.204) (Table 1, Additional file 1: Table S1). Likewise, no significant difference was observed between gliomas WHO II and III (*p* = 0.194).

#### Correlation of LAT1 overall H-score with the ^18^F-FET uptake

**Fig. 2:**
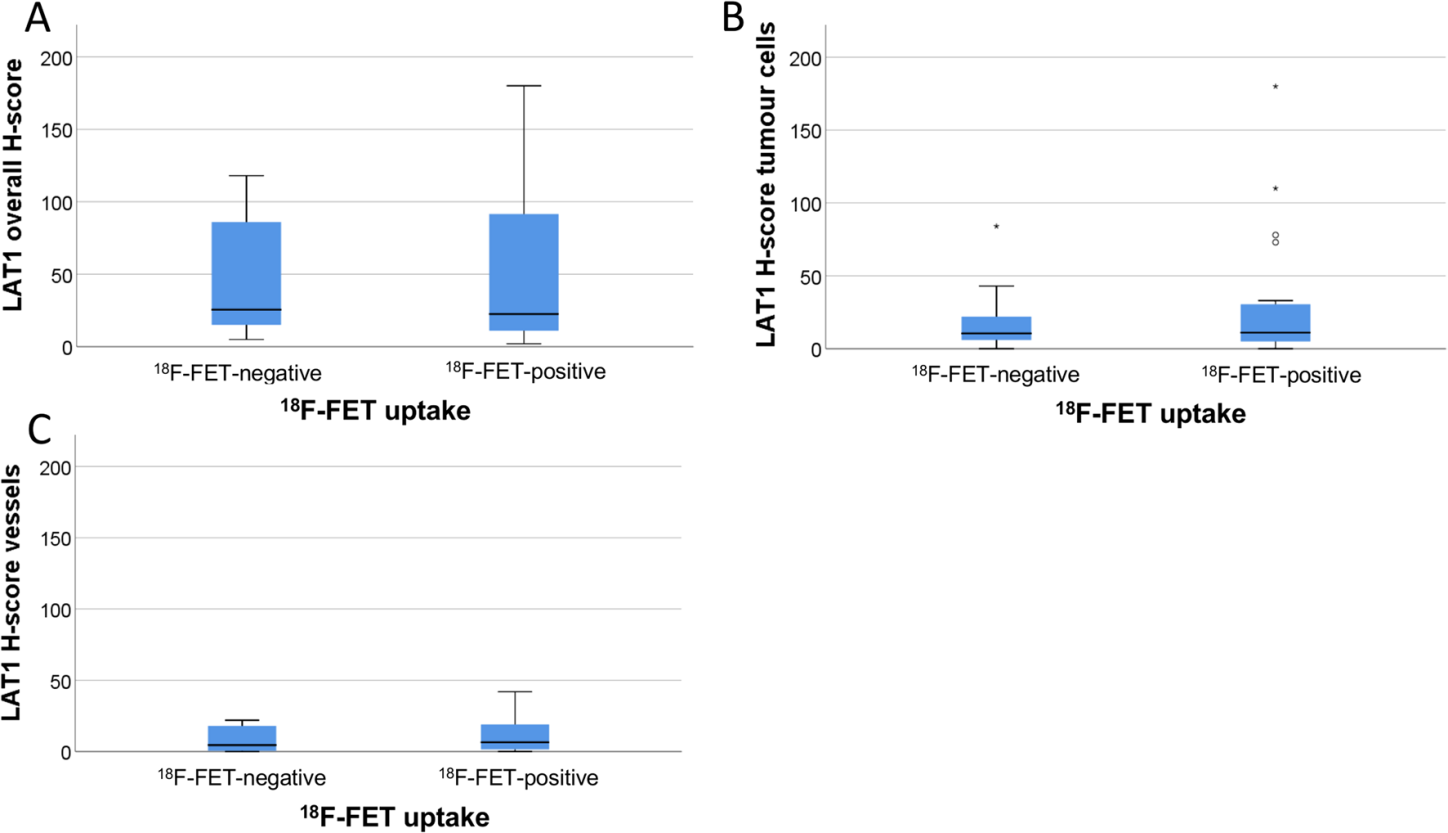
LAT1 overall H-score (**A**), the LAT1 H-score tumour cells (**B**) and the LAT1 H-score vessels (**C**) in the ^18^F-FET-negative and ^18^F-FET-positive gliomas

^18^F-FET-negative gliomas showed a median H-score of 25.5 (range 5–118) compared to ^18^F-FET-positive gliomas with a median H-score of 22.5 (range 2–180) (Fig. 2a). We found no significant correlation between the overall H-score and the two ^18^F-FET uptake groups (*p* = 0.832).

#### Correlation of LAT1 H-score tumour cells with the ^18^F-FET uptake

Scoring the tumour cells only, excluding staining from contaminating cell populations, non-neoplastic tissue or endothelial staining, no correlation between the H-score and the two ^18^F-FET uptake groups was detectable (*p* = 0.204). ^18^F-FET-negative gliomas showed a median H-score of 10.5 (range 0–84) and ^18^F-FET-positive gliomas a median H-score of 11 (0–180) (Fig. 2b).

In the subgroup of stereotactically biopsied patients the correlation between the H-score and the ^18^F-FET uptake groups was also not significant (*p* = 0.797).

#### Correlation of LAT1 H-score vessels with the ^18^F-FET uptake

In line with the findings above, the H-score of endothelial staining showed no significant difference between the two ^18^F-FET uptake groups (*p* = 0.298). The overall amount of vessels in most of the stereotactically obtained specimen was rather low.

^18^F-FET-negative gliomas showed a median H-score of 4.5 (range 0–22) and ^18^F-FET-positive gliomas a median H-Score of 6.5 (0–42) (Fig. 2c).

#### Correlation of TBR_max_ with LAT1 expression

**Fig. 3:**
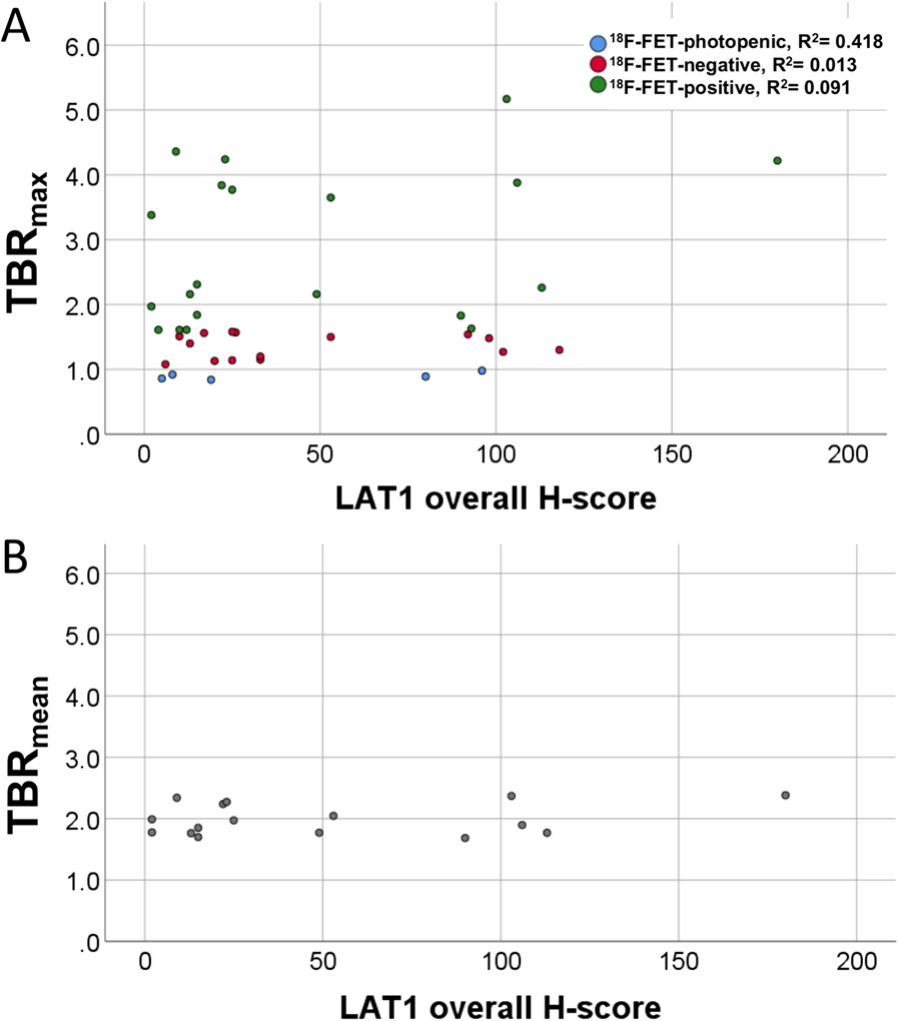
Distribution of the TBR_max_ (**A**) and TBR_mean_ (**B**) over the LAT1 overall H-score in the three groups of ^18^F-FET-photopenic, ^18^F-FET-negative and ^18^F-FET-positive gliomas: no correlation was found for the LAT1 overall score and the ^18^F-FET-PET parameter. *R*^2^ values are displayed in the legend (**A**)

No significant correlation of the TBR_max_ with the overall H-score of LAT1 expression was detectable (*p* = 0.201, Fig. 3a). Likewise, there was no significant correlation between the LAT1 H-score tumour cells with the TBR_max_ (*p* = 0.365).

#### Correlation of TBR_max_ and TBR_mean_ with LAT1 expression in ^18^F-FET positive gliomas only

Similarly, TBR_max_ did not correlate with the LAT1 expression in the ^18^F-FET-positive subgroup (*p* = 0.301). In ^18^F-FET-positive gliomas, TBR_mean_ was assessed as described above (21) and did also not correlate with the LAT1 expression (*p* = 0.154, Fig. 3b).

#### Correlation of TTP_min_ with LAT1 expression in ^18^F-FET positive gliomas only

No significant correlation of TTP_min_ and LAT1 expression in the ^18^F-FET-positive subgroup (*p* = 0.267) was detectable.

### Outcome analysis

The median follow-up time was 35 months (range 9–110 months). During the follow-up-time, 29 of 40 patients had experienced tumour progression. The median PFS was 29.0 months (range 9–84 months).

#### Survival analysis of high versus low LAT1 expression

Univariate analysis revealed the level of LAT1 expression (overall H-score) to be a significant prognostic marker for PFS (*p* = 0.033).

**Fig. 4:**
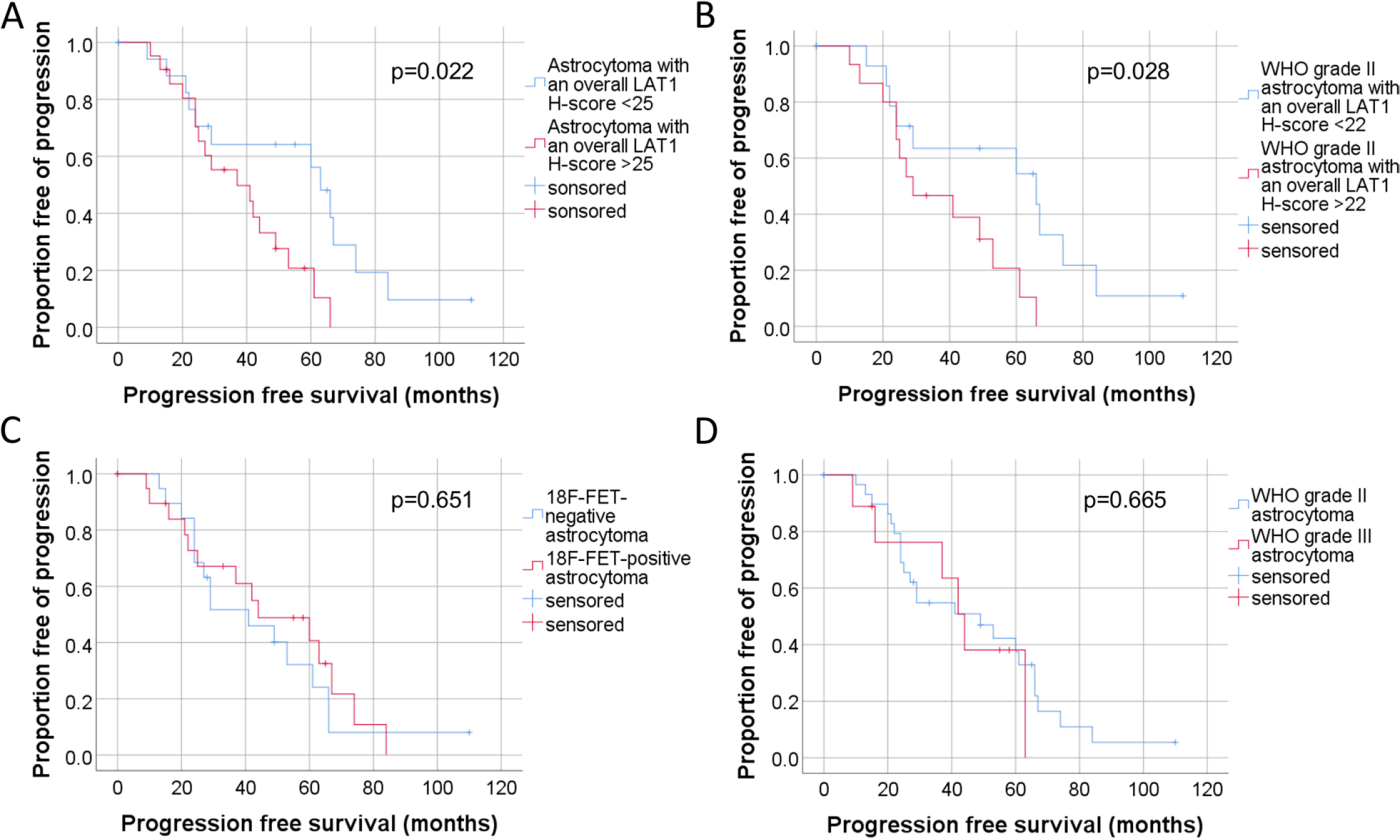
Survival analysis of the LAT1 expression in the overall group (**A**), in WHO grade II astrocytomas (**B**), of ^18^F-FET-positive versus ^18^F-FET-negative astrocytomas (**C**) and of the WHO grade II and III astrocytomas (**D**)

The Kaplan–Meier analysis with log-rank test revealed a significant difference in the PFS of patients with high versus low LAT1 expression. The median split was calculated at the overall H-score of 25. Patients with an initially higher LAT1 expression (H-score > 25) showed a significantly shorter median PFS with 37.0 months (95% CI 13.0–61.0 months) compared to patients with a lower LAT1 expression (H-score < 25) with a PFS of 63.0 months (95% CI 54.2–71.8 months; Chi-Quadrat 5.273; *p* = 0.022) (Fig. 4a).

To exclude any influence of the nine WHO grade III gliomas, we performed the survival analysis with exclusion of WHO grade III patients. The median split in the subgroup of WHO grade II gliomas was an H-score of the overall LAT1 expression of 22. Results were comparable to the overall group with a significant correlation of the PFS with LAT1 expression (*p* = 0.028). WHO grade II astrocytomas with an initially higher H-score (> 22) showed a significantly shorter median PFS of 29.0 months (95% CI 9.9–48.2 months) compared to the WHO grade II astrocytomas with a lower H-score (< 22) with a median PFS of 66 months (95% CI 12.1–120.0 months; Chi-Quadrat 4.834; *p* = 0.028) (Fig. 4b).

#### Influence of the ^18^F-FET uptake on survival

Comparing the different survival data of the ^18^F-FET uptake groups, no significant correlation was detectable. In the univariate analysis ^18^F-FET-uptake (TBR_max_) showed no significant prognostic relevance for the PFS in the overall group (*p* = 0.655).

The median PFS of patients with an ^18^F-FET-positive astrocytoma was 44.0 months (95% CI 17.3–70.7 months) compared to the median PFS of patients with an ^18^F-FET-negative astrocytoma of 41.0 months (95% CI 19.1–63.0 months; Chi-Quadrat 0.205; *p* = 0.651) (Fig. 4c).

#### Influence of the WHO grade on survival

The progression-free survival did not differ between the gliomas with WHO grades II or III in our cohort. The median PFS of patients with a WHO grade II glioma was 49 months (95% CI 24.6–73.4 months) compared to the median PFS of patients with a WHO grade III astrocytoma of 44.0 months (95% CI 34.7–53.3 months; Chi-Quadrat 0.188; *p* = 0.665) (Fig. 4d).

## Discussion

The pathophysiologic mechanisms leading to ^18^F-FET uptake and the different dynamic uptake characteristics are not yet fully clarified, though it is suggested that the L-type amino acid transporter 1 is primarily responsible for the uptake. However, it remains unclear which cellular mechanisms lead to the phenomenon of missing or lower intracellular ^18^F-FET uptake in ^18^F-FET-negative gliomas. This study systematically evaluates the LAT1 expression of ^18^F-FET-positive and ^18^F-FET-negative gliomas within a homogeneous group of newly diagnosed *IDH*-mutant astrocytomas with a large number of histopathological samples. Additionally, LAT1 expression was investigated concerning its prognostic influence.

It has been suggested that ^18^F-FET-negative or ^18^F-FET-photopenic gliomas may lack the typical overexpression of LAT1 and thus not be able to show enhanced ^18^F-FET uptake [17]. However, in our homogenous cohort of WHO grade II and III, *IDH*-mutant astrocytomas without 1p/19q codeletion ^18^F-FET-negative gliomas were consistently positive in LAT1 staining. Not even a quantitative comparison with the ^18^F-FET-positive gliomas detected a significant difference of LAT1 expression levels.

Consistent with our results, Stockhammer et al. found no correlation between LAT1 staining intensity and ^18^F-FET uptake in a similar cohort with only non-contrast enhancing WHO grade II and III astrocytomas and oligodendrogliomas [30]. Notably, Stockhammer et al. reported a rather low staining intensity, which is in line with our findings. Although Habermeier et al. showed a predominant ^18^F-FET accumulation via LAT1 in human LN229 glioblastoma cells [7] and Youland et al. reported LAT1 as a key determinant of ^18^F-DOPA accumulation in glioblastoma cell lines [4], the observed low staining intensity may indicate that LAT1 may not be the primary transporter in *IDH*-mutant WHO grade II and III gliomas [30]. Okubo et al. reported lower LAT1 expression in low-grade compared to high-grade gliomas and an increasing level of LAT1 immunostaining with higher glioma grades [31], which may also explain the generally low staining intensity in our patients.

Lahoutte et al. reported that ^18^F-FET is only a poor uptake substrate for LAT1 in Xenopus laevis oocytes, although the experimental setting using oocytes does not fully reflect the situation of in vivo tumour imaging in humans [2]. Based on this study and studies about ^18^F-FET accumulation in rat F98 glioma cells, it has been discussed if ^18^F-FET may be selectively transported via a different L-type amino acid transporter 2 [6]. However, a subsequent study performing competition studies on Xenopus laevis oocytes expressing mouse LAT2/4F2hc found a rather low affinity of LAT2 for ^18^F-FET [32].

The literature to date is contradictory as to which of the LAT transporters are primarily responsible for the ^18^F-FET uptake. This may be due to the heterogeneous groups of glioma entities that were evaluated in previous studies, which may not be comparable, and there might be a difference in the transporter system or transporter expression in different glioma subtypes.

Further studies should investigate homogeneous glioma groups and additionally analyse LAT2 expression levels and the complex SLC-transporter family in more detail.

To further evaluate the level of LAT1 expression and possible methylation differences, we performed a subanalysis of the LAT1 promoter data from TCGA (The Cancer Genome Atlas) looking for alterations in LAT1 promoter methylation. Interestingly, these data provide evidence that the *LAT1* promoter may be silenced by promoter methylation in gliomas (explaining for the overall low LAT1 protein expression levels) with the degree of promoter methylation appearing even higher in the TCGA low-grade compared to the TCGA GBM cohort. Differential methylation in individual glioma subgroups may also explain contradictory results in the literature, due to heterogeneous cohorts which have been studies and compared. Likewise Linzhi Cai et al. performed a TCGA dataset analysis for the gene expression of LAT1 over several glioblastoma subtypes but did not find a significant variation in the LAT1 expression in the different genetic subtypes [5], noting only different glioblastoma subtypes were compared.

It has been proposed that glioma patients with low amino acid uptake have a favourable prognosis with a lower risk for tumour relapse and malignant progression within the first 5 years after initial diagnosis [33]. In line with our findings though, several other studies could not confirm these results and did not find any significant difference in the outcome [17, 22, 34]. This discrepancy may be due to heterogeneous cohorts without consideration of molecular genetic subgroups determining malignancy and resulted in an intercorrelation of prognostically relevant factors and ^18^F-FET uptake.

Recent studies have turned focus on a third subgroup of gliomas with lower ^18^F-FET-uptake than the physiological brain tissue—namely photopenic lesions in gliomas. Interestingly, this subgroup does not only show adverse performance in clinical outcome compared to isometabolic ^18^F-FET-negative gliomas with a shorter progression-free survival, but even seems to have a more aggressive clinical behaviour than neuropathologically matched ^18^F-FET-positive gliomas [17].

Interestingly in analogy to ^18^F-FET, the negative prognostic factor has also been reported in ^18^F-DOPA photopenic gliomas, but no studies have yet reasonably explained this phenomenon [35]. The impact of PET-negativity has already been described for other amino acid tracer, such as ^18^F-DOPA and ^11^C-MET, especially in low-grade gliomas with comparable frequencies in all three tracers, without further research on this topic [36, 37].

Due to only five ^18^F-FET-photopenic lesions in our cohort, a detailed statistical analysis was not feasible, though ^18^F-FET-photopenic lesions were visually positive on LAT1 staining and no quantitative difference from ^18^F-FET-positive gliomas was observed, at least in this small group.

Basic research has increasingly highlighted that LAT1 plays a prominent role in malignancy, showing a close correlation of LAT1 overexpression and malignant phenotype as well as cancer cell growth and proliferation [10]. The main function of the LAT1 protein is to help specific amino acids pass through the cell membrane to provide nutrients to cells and participate in metabolic pathways. LAT1 is over-expressed in many human cancer cells that are characterized by an increased demand of essential amino acids including gliomas [11–15]. The upregulated expression of LAT1 may benefit by providing tumour cells with essential amino acids for high levels of protein synthesis associated with cell activation to support rapid growth or excessive proliferation. Additionally, the LAT1 level in tumours was recently shown to be an independent prognostic indicator for malignant progression and proliferation of high-grade gliomas and correlates closely with the glioma angiogenesis [10, 16]. High LAT1 expression is associated with poor prognosis and shorter progression-free survival in various cancers (e.g. ovarian cancer, pancreatic cancer, adenoid, cystic cancer, lung neuroendocrine tumours) [38–45]. For glioma, fewer reports exist regarding the precise prognostic role of LAT1. The related data indicate that LAT1 is associated with fast progression and poor prognosis [10, 16, 46], although these studies only regarded glioblastomas [47].

This is the first study to investigate LAT1 expression in the homogeneous group of WHO grade II and III *IDH*-mutant astrocytomas, reporting an association of higher LAT1 expression with faster progression. Our data fit with previous findings in the literature for gliomas and other cancers regarding the highly prognostic value of LAT1 [16, 39, 45]. Of note, the prognostic value of LAT1 expression remained significant even in the subgroup of WHO grade II gliomas only. Interestingly, the LAT1 expression levels were not associated with the WHO grade, although WHO grade III tumours were found among ^18^F-FET-positive gliomas only. This is particularly interesting, as neither WHO grade nor ^18^F-FET-uptake intensity correlated with the PFS, while LAT1 expression did. One might therefore hypothesize that a so far unknown biological process that is associated with the histological tumour grade may have an impact on ^18^F-FET-uptake intensity in gliomas, but without being related to LAT1 expression level or outcome [34]. This would also be in line with a finding of a previous study, where we found that a switch from a ^18^F-FET-negative to a ^18^F-FET-positive glioma was associated with a malignant transformation from WHO grade II to WHO grade III tumour.

The following limitations of the current study need to be addressed. The study is based on retrospective data, and the results need to be confirmed in a prospective study design. Biopsies may potentially be subject to sampling errors, although ^18^F-FET-negative gliomas were ^18^F-FET-negative throughout the whole tumour, so the specific location of the biopsy within the tumour in unlikely to have an impact. The specimens would have been expected to be completely LAT1-negative, but this was not the case. In ^18^F-FET-positive gliomas, a biopsy is usually performed based on a ^18^F-FET PET guided hot spot, so sampling should reflect the area of maximal uptake. It may be considered as another limitation of our study that effects on overall survival have not been analyzed. This, however, is due to the circumstance that a significant number of events have not yet occurred with regard to overall survival, which lays in the natural course of disease in WHO grade II/III *IDH*-mutant astrocytomas. We will follow up on this aspect over time. The LAT1 transporter complex consists of two parts, the light chain LAT1 and the heavy chain 4F2hc, and it is a major limitation of this study that only the expression of LAT1 could be determined. Therefore, the results of this study have to be interpreted with caution although 4F2hc is assumed to be strongly positively correlated to the expression level of LAT1 [10]. Nevertheless, the finding of high ^18^F-FET uptake in some tumours showing low levels of LAT1 expression (Fig. 1) raises some doubt on the role of LAT1 in 18F-FET uptake, which needs further investigation.

## Conclusion

Although LAT1 is reported to mediate the uptake of ^18^F-FET into tumour cells, the levels of LAT1 expression do not correlate with the levels of ^18^F-FET uptake in a homogeneous group of *IDH*-mutant astrocytomas WHO grade II/III. In particular, the lack of tracer uptake in ^18^F-FET-negative gliomas cannot be explained by reduced levels of LAT1 expression. A higher LAT1 expression as assessed by immunohistochemistry seems to be associated with a short PFS in *IDH*-mutant astrocytomas. Further studies regarding mechanisms influencing the uptake of ^18^F-FET are necessary to elucidate the phenomenon of missing ^18^F-FET uptake in ^18^F-FET-negative gliomas.

## Supplementary Information

**Additional file 1**. Full patients characteristics with detailed LAT1 H-score values.

## Data Availability

The datasets used and analysed during the current study are available from the corresponding author on reasonable request.
