# Supplementary material for: L-type amino acid transporter (LAT) 1 expression in 18F-FET-negative gliomas
Source: EJNMMI Res. 2021 Dec 14;11:124. doi: 10.1186/s13550-021-00865-9 (PMC8671595; doi:10.1186/s13550-021-00865-9)
Supplement: Supplementary file 1 — Additional file 1. Full patients characteristics with detailed LAT1 H-score values. [file 13550_2021_865_MOESM1_ESM.docx]

Supplements

Supplement table 1

| # | age  (years) | sex | mode of  surgery | WHO grade | FET uptake | TBR_max_ | LAT1 overall  H-score | LAT1 H-score tumour cells | LAT1 H-score vessels | progression | Progression free survival (months) |
| --- | --- | --- | --- | --- | --- | --- | --- | --- | --- | --- | --- |
| 1 | 40.99 | m | biopsy | 2 | negative | 1.50 | 53.00 | 34.00 | 1.00 | yes | 24 |
| 2 | 26.25 | m | biopsy | 2 | negative | 1.57 | 26.00 | 25.00 | 4.00 | yes | 61 |
| 3 | 43.08 | m | biopsy | 2 | negative | 0.84 | 19.00 | 9.00 | 4.00 | yes | 29 |
| 4 | 47.33 | f | resection | 2 | negative | 0.86 | 5.00 | 0.00 | 0.00 | no | 49 |
| 5 | 37.26 | m | biopsy | 2 | negative | 1.56 | 17.00 | 13.00 | 0.00 | yes | 24 |
| 6 | 35.77 | m | biopsy | 2 | negative | 0.92 | 8.00 | 8.00 | 2.00 | yes | 15 |
| 7 | 47.54 | m | biopsy | 2 | negative | 1.20 | 33.00 | 13.00 | 0.00 | yes | 29 |
| 8 | 41.68 | f | biopsy | 2 | negative | 1.14 | 25.00 | 21.00 | 8.00 | yes | 24 |
| 9 | 30.30 | m | biopsy | 2 | negative | 1.27 | 102.00 | 12.00 | 18.00 | yes | 27 |
| 10 | 39.45 | f | biopsy | 2 | negative | 1.13 | 20.00 | 9.00 | 13.00 | no | 110 |
| 11 | 27.43 | f | biopsy | 2 | negative | 1.30 | 118.00 | 84.00 | 21.00 | yes | 13 |
| 12 | 27.00 | f | biopsy | 2 | negative | 1.48 | 98.00 | 5.00 | 18.00 | yes | 41 |
| 13 | 38.29 | m | biopsy | 2 | negative | 1.58 | 25.00 | 23.00 | 4.00 | yes | 49 |
| 14 | 30.51 | m | biopsy | 2 | negative | 1.08 | 6.00 | 12.00 | 5.00 | no | 28 |
| 15 | 37.29 | f | biopsy | 2 | negative | 1.15 | 33.00 | 43.00 | 0.00 | yes | 53 |
| 16 | 44.22 | m | biopsy | 2 | negative | 1.51 | 10.00 | 2.00 | 0.00 | no | 0 |
| 17 | 44.86 | f | biopsy | 2 | negative | 0.89 | 80.00 | 5.00 | 10.00 | yes | 66 |
| 18 | 47.60 | f | biopsy | 2 | negative | 0.98 | 96.00 | 7.00 | 18.00 | yes | 20 |
| 19 | 27.71 | m | biopsy | 2 | negative | 1.54 | 92.00 | 5.00 | 22.00 | no | 49 |
| 20 | 28.67 | f | biopsy | 2 | negative | 1.40 | 13.00 | 8.00 | 22.00 | yes | 66 |
| 21 | 53.86 | f | biopsy | 2 | positive | 1.61 | 12.00 | 27.00 | 0.00 | yes | 21 |
| 22 | 26.23 | m | resection | 3 | positive | 4.22 | 180.00 | 180.00 | 0.00 | yes | 42 |
| 23 | 34.23 | f | biopsy | 3 | positive | 2.16 | 49.00 | 13.00 | 0.00 | no | 58 |
| 24 | 49.27 | m | biopsy | 2 | positive | 3.88 | 106.00 | 28.00 | 28.00 | yes | 25 |
| 25 | 35.75 | m | biopsy | 2 | positive | 2.16 | 13.00 | 8.00 | 5.00 | yes | 22 |
| 26 | 69.14 | f | biopsy | 2 | positive | 1.61 | 10.00 | 2.00 | 5.00 | yes | 67 |
| 27 | 31.29 | m | biopsy | 3 | positive | 4.24 | 23.00 | 2.00 | 0.00 | yes | 9 |
| 28 | 41.64 | m | biopsy | 3 | positive | 2.26 | 113.00 | 12.00 | 42.00 | no | 15 |
| 29 | 38.28 | m | biopsy | 2 | positive | 5.17 | 103.00 | 73.00 | 2.00 | yes | 10 |
| 30 | 32.17 | f | biopsy | 2 | positive | 1.63 | 93.00 | 9.00 | 9.00 | no | 33 |
| 31 | 25.88 | m | resection | 3 | positive | 3.77 | 25.00 | 33.00 | 20.00 | yes | 44 |
| 32 | 29.27 | f | resection | 3 | positive | 1.84 | 15.00 | 15.00 | 40.00 | yes | 63 |
| 33 | 46.19 | m | resection | 3 | positive | 3.38 | 2.00 | 5.00 | 10.00 | no | 55 |
| 34 | 34.81 | f | biopsy | 2 | positive | 4.36 | 9.00 | 5.00 | 5.00 | yes | 60 |
| 35 | 51.43 | m | biopsy | 3 | positive | 3.65 | 53.00 | 78.00 | 13.00 | yes | 16 |
| 36 | 42.26 | f | biopsy | 2 | positive | 1.61 | 4.00 | 0.00 | 1.00 | yes | 84 |
| 37 | 34.25 | m | biopsy | 2 | positive | 2.31 | 15.00 | 10.00 | 18.00 | yes | 74 |
| 38 | 69.31 | m | biopsy | 2 | positive | 3.84 | 22.00 | 10.00 | 8.00 | no | 0 |
| 39 | 46.10 | f | resection | 3 | positive | 1.83 | 90.00 | 110.00 | 35.00 | yes | 37 |
| 40 | 56.27 | m | resection | 2 | positive | 1.97 | 2.00 | 5.00 | 5.00 | no | 65 |
